# A case of immunotherapy-induced thyroiditis

**DOI:** 10.37349/etat.2024.00214

**Published:** 2024-02-28

**Authors:** George Pears, Abhishek Mahajan, Anna Olsson-Brown, Joseph Sacco

**Affiliations:** Kumamoto University, Japan; ^1^Department of Imaging, The Clatterbridge Cancer Centre NHS Foundation Trust, L7 8YA Liverpool, UK; ^2^Faculty of Health and Life Sciences, University of Liverpool, L7 8TX Liverpool, UK; ^3^Oncology, The Clatterbridge Cancer Centre NHS Foundation Trust, L7 8YA Liverpool, UK

**Keywords:** Immunotherapy, thyroiditis, melanoma, cancer, radiology, endocrinology

## Abstract

Immunotherapy treatments for cancer are known to cause adverse thyroid events which present a diagnostic challenge to clinicians and radiologists. This case report highlights the importance of a high clinical index of suspicion and careful assessment of the thyroid on serial imaging studies to make the diagnosis. The case involves a 65-year-old male with malignant melanoma who was started on immunotherapy as part of a clinical trial. He developed thyroid dysfunction followed by an attack of acute neck pain. Ultrasound of his thyroid was performed which showed significant atrophy. A review of previous imaging was undertaken which confirmed the patient had suffered from thyroiditis and subsequent atrophy. Following this, the diagnosis of immunotherapy-induced thyroid dysfunction was made. Thyroxine supplementation and steroid dose were then adjusted causing his thyroid function and symptoms to improve. Immunotherapy agents for cancers are becoming more and more common. As the case report shows, physicians and radiologists will need to be vigilant to diagnose and treat any adverse events.

## Introduction

Melanoma is the 5th most common cancer in the United Kingdom (UK) with 16,700 new cases diagnosed every year and it accounts for 1% of all cancer deaths [1]. Immunotherapy is an effective treatment due to the high immunogenicity of melanoma. Recent studies have shown a significantly higher success rate with a combination of immunotherapy and chemotherapy, radiotherapy, or targeted molecular therapy [2]. In the UK, immunotherapy is used to treat patients with advanced metastatic (stage IV) melanoma and unresectable stage III disease [3]. They are also commonly used agents in other cancers such as metastatic renal cell, non-small cell lung, hepatocellular and bladder. Immunotherapy agents are revolutionising melanoma and other cancer treatments and are becoming more widely used. However, they cause significant adverse events to multiple organ systems. Examples include endocrine disturbances, hepatitis, pneumonitis, colitis and dermatitis [4].

The thyroid gland is an organ that is commonly affected but diagnosis can often be delayed or missed due to a lack of clear signs and symptoms. It is an important condition to diagnose as it can lead to significant morbidity for patients. This case report of immunotherapy-induced thyroiditis in a 65-year-old male with malignant melanoma aims to highlight the role of clinical, laboratory and radiological factors in diagnosis. It will outline how a high index of clinical suspicion can facilitate a prompter diagnosis. The case will also aim to show how radiology, specifically careful analysis of serial imaging studies, can contribute to the diagnosis of this important condition.

## Case report

### Clinical history

The case involves a 65-year-old male diagnosed with tumour stage 4b, nodal stage unknown and metastatic stage 1b (T4bNxM1b) malignant melanoma of the upper back. He started a combination of ipilimumab and nivolumab in March 2020. He then had biopsy-proven skin recurrence in September 2021 and was started on nivolumab again in November 2021 as part of the RP2 clinical trial (NCT04336241).

Between April-August 2022 the patient’s blood analysis revealed persistently high thyroid stimulating hormone (TSH) up to 46 mU/L (normal reference range 0.27–4.20) and triiodothyronine (T3)/thyroxine (T4) levels as low as 2.6 pmol/L and 3.5 pmol/L respectively (normal reference ranges 3.1–6.8 and 12.0–22.0 respectively). Please see Table 1 for serial analysis. On questioning, the patient was suffering from symptoms of hypothyroidism. Immune-related indices were performed as part of the patient’s workup and were shown to be negative with results as follows: thyroid receptor antibody < 1.1 IU/L (negative), thyroglobulin antibody 12 kIU/L (normal < 20 kIU/L) and thyroid peroxidase antibody 9 kIU/L (normal < 30 kIU/L). Due to clinical concerns regarding thyroid dysfunction, the patient was started on 75 mcg/day of levothyroxine in May 2022. As you can see from Table 1, there was some improvement in TSH following levothyroxine initiation, but the thyroid function tests (TFTs) remained deranged. It is unclear at this point whether immunotherapy being the causative agent of thyroid dysfunction was considered.

**Table 1:** Serial TFT results pre-diagnosis

**Date**	**TSH (mU/L; normal reference range 0.27–4.20)**	**T3 (pmol/L; normal reference range 3.1–6.8)**	**T4 (pmol/L; normal reference range 12.0–22.0)**
25/04/22	46	2.6	3.5
23/05/22	43	2.6	6.5
04/07/22	15.5	2.8	8.4
01/08/22	19.8	2.6	9.7

The 25/04/22 means 25th April 2022; other dates are in the same format

The patient subsequently presented with acute anterior neck pain on the 12th of August 2022 and an urgent thyroid ultrasound was performed.

### Imaging findings

**Figure 1:**
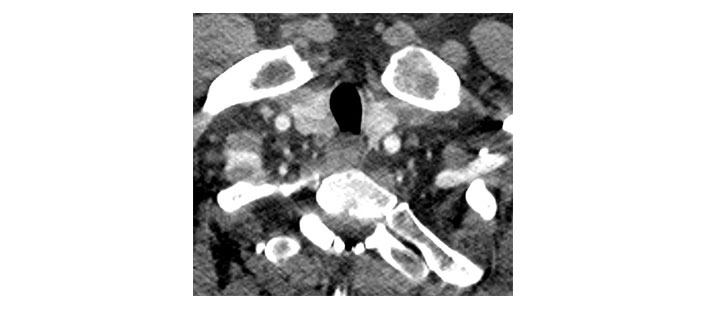
Axial CT image dated 2nd September 2020

**Figure 2:**
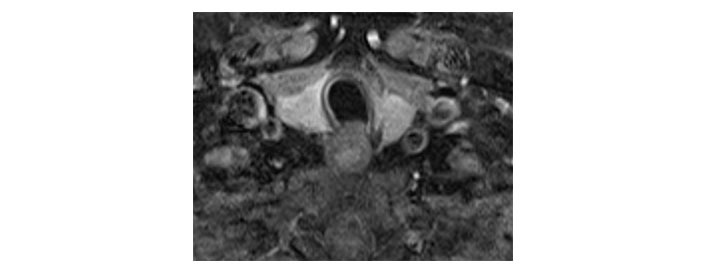
Axial T1 fat-saturated post-contrast image dated 28th October 2021

**Figure 3:**
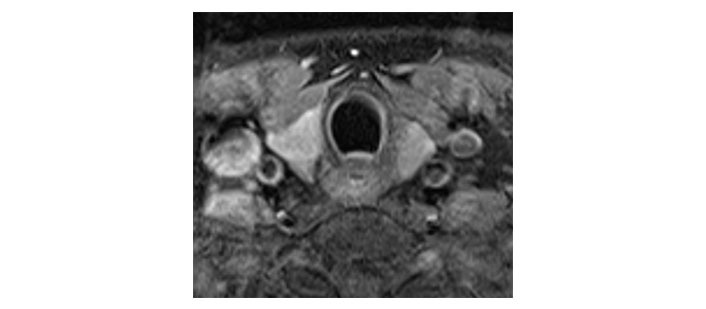
Axial T1 fat-saturated post-contrast image dated 7th January 2022

**Figure 4:**
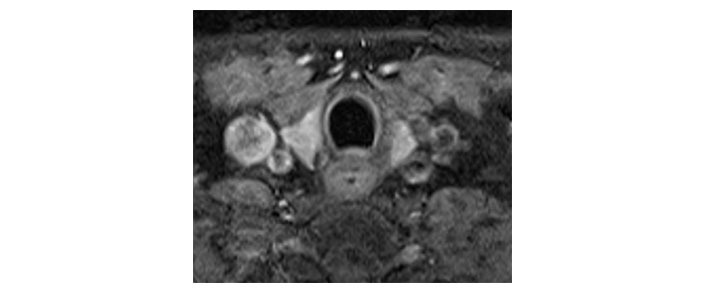
Axial T1 fat-saturated post-contrast image dated 15th May 2022

**Figure 5:**
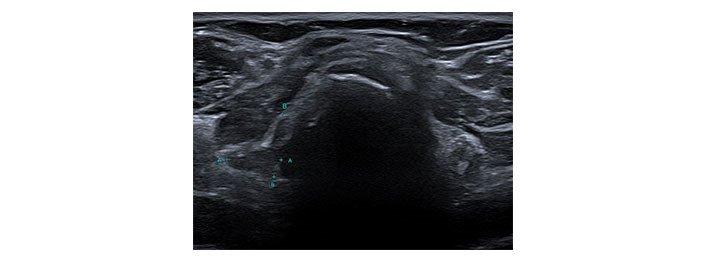
Transverse ultrasound image of the thyroid dated 12th August 2022. Callipers on the right lobe measured approximately 12 mm × 11 mm. A: transverse diameter of the right lobe of thyroid = 11mm; B: longitudinal diameter of the right lobe of thyroid = 12mm

**Figure 6:**
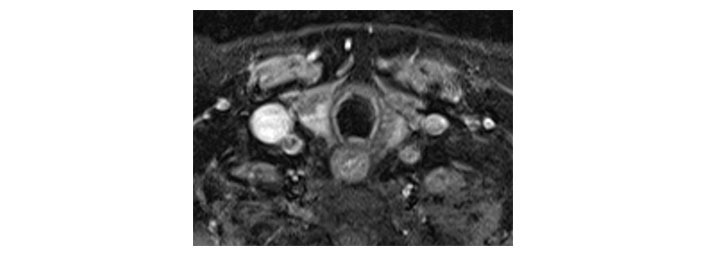
Axial T1 fat-saturated post-contrast image dated 17th August 2022

Please see Figures 1, 2, 3, 4, 5, and 6 for serial radiological studies of the patient’s thyroid gland. Computed tomography (CT) performed in September 2020 (Figure 1) and magnetic resonance imaging (MRI) performed in October 2021 (Figure 2) show normal thyroid volume. As detailed in the clinical history, the patient started immunotherapy in November 2021. An MRI study performed in January 2022 (Figure 3) shows a slight reduction of the thyroid volume (most apparent on the left side). MRI in May 2022 (Figure 4) shows further reduction in the thyroid volume which can be appreciated in both lobes. Up to this point the reduced thyroid volume had not been appreciated on imaging and the patient had not been formally diagnosed with thyroiditis. The patient presented with acute anterior neck pain in August 2022 so an ultrasound thyroid was performed (Figure 5) which showed significantly reduced thyroid volume. An MRI study also performed in August 2022 (Figure 6) shows similar findings. The findings on the August 2022 studies prompted a retrospective review of previous imaging and the gradual reduction in thyroid volume was then appreciated. The combination of this, the patient’s symptoms and their TFTs led to a diagnosis of immunotherapy-induced thyroiditis. This shows the importance of assessing the thyroid in serial imaging studies in these patients. Only with careful retrospective review could the atrophy be fully appreciated and contribute to the patient’s diagnosis.

### Diagnosis and treatment

Analysis of the imaging detailed above, the patient’s clinical symptoms and the patient’s TFTs led to a formal diagnosis of immunotherapy-induced thyroiditis. Following this, the patient’s levothyroxine was increased to 100 mcg/day and their steroid dose increased. The immunotherapy regime was continued and not adjusted.

The patients’ TFTs following diagnosis and adjustment of steroid and levothyroxine dosages are shown in Table 2. As you can see, in December 2022 their TFTs had normalised which is a stark contrast to the result from 1st August 2022 in Table 1 (which was before the formal diagnosis of immunotherapy-induced thyroiditis was made and before their treatment was adjusted). Between 1st August 2022 and 12th December 2022 the TSH improved from 19.8 mU/L to 3.2 mU/L and the T4 levels improved from 9.7 pmol/L to 15.4 pmol/L. Their clinical symptoms also improved following diagnosis and treatment adjustment.

**Table 2:** Serial TFT results post-diagnosis and following steroid and levothyroxine dose adjustment

**Date**	**TSH (mU/L; normal reference range 0.27–4.20)**	**T3 (pmol/L; normal reference range 3.1–6.8)**	**T4 (pmol/L; normal reference range 12.0–22.0)**
30/08/22	6.6	3.6	13.4
24/10/22	3.6	2.9	14.8
02/11/22	1.4	3.7	11.5
12/12/22	3.2	3.9	15.4

## Discussion

Immunotherapy agents focus on specific targets of the immune system’s counter-regulatory mechanisms. Although these agents are more targeted and therefore better tolerated compared to most chemotherapy agents, they are associated with a new group of specific immune-related adverse events (irAEs) of which one group is endocrine [2]. Endocrine toxicities include hyperthyroidism, hypothyroidism, thyroiditis, hypophysitis and adrenal insufficiency. They usually appear after 6 weeks of treatment and may take a long time to resolve, in some cases being irreversible. These toxicities are important to detect as they can lead to significant morbidity and severe electrolyte imbalances. However, they present a diagnostic challenge as they can often present insidiously with vague symptoms. A combination of clinical, biochemical and radiological factors is necessary for diagnosis [4].

Whilst scholars believe most patients on immunotherapy are at risk of thyroid irAEs, their exact incidence is not known and depends on the specific agent being used [5]. Thyroid irAEs seem to have some overlap with Hashimoto’s thyroiditis, Grave’s disease and other autoimmune thyroid diseases. The precise relationship between thyroid irAEs and these autoimmune diseases remains to be seen [5].

Clinical manifestations of thyroid irAEs are notably variable with some patients experiencing hypothyroidism and others transient thyroiditis. Patients are mostly detected during routine hormone monitoring and screening [6]. Indeed, the diagnosis of thyroid dysfunction is predominately based on TFTs, even in the absence of clinical symptoms. TFTs should be part of baseline laboratory testing in all patients undergoing treatment with immunotherapies and should be regularly monitored. However, interpretation should be undertaken with caution as numerous factors may affect the hypothalamic-pituitary-thyroid axis in cancer patients (such as steroids, non-thyroid illnesses and iodinated contrast injection) [7].

Information regarding imaging recommendations and findings in thyroid irAEs is sparse. Ultrasound provides an inexpensive, dynamic and extremely useful assessment of the thyroid to aid in diagnosis. During an active phase of thyroiditis, the gland can appear large and heterogeneous with increased vascularity. Any subsequent atrophy and reduced activity (with associated reduced vascularity) will be demonstrated [7]. It is known that active thyroiditis can be associated with increased 2-deoxy-2-[^18^F] fluoro-d-glucose (^18^FDG) positron emission tomography (PET)/CT activity when compared with baseline studies, especially when TFTs are abnormal [5]. Neck CT can also reveal abnormalities of thyroiditis such as diffuse enlargement of the gland with hypoattenuation and occasional areas of ring enhancement from gland necrosis. Once the active phase of the disease has resolved gland atrophy and subsequent hypothyroidism can develop with CT and PET/CT findings reflective of this [8].

Treatment of immunotherapy-induced thyroiditis depends on the severity and clinical/biochemical manifestations. Patients with hypothyroidism should be treated with continuous T4. Transient hyperthyroidism should be treated conservatively as it often subsides and transforms into hypothyroidism. However, patients with signs of severe thyrotoxicosis should be treated aggressively with a combination of supportive therapies (beta blockers and glucocorticoids) and anti-thyroid drugs to help relieve symptoms and avoid complications [6].

Fortunately, new agents are being developed to counter immunotherapy toxicities. For example, nanomaterial-based immunotherapies such as stimuli-responsive nanoparticles offer an exciting future. Emerging evidence shows positive outcomes in tumour response whilst adverse events are kept to a minimum when compared with immunotherapy alone [9, 10]. Once widespread utilisation of these treatment methods is adopted the burden of adverse events on patients should be reduced.

In conclusion, thyroid irAEs from immunotherapy treatments are an important cause of morbidity in cancer patients and present a diagnostic challenge to clinicians and radiologists. As the case report shows, a high index of clinical suspicion is important and could have aided earlier diagnosis, therefore in cancer patients suffering from thyroid dysfunction, the causative relationship with any immunotherapy treatments should always be considered. The case report also highlights how careful assessment of the thyroid in serial imaging studies can help detect any subtle evolving changes to aid early diagnosis and treatment.

## Abbreviations

CT: computed tomography
irAEs: Immune-Related Adverse Events
MRI: magnetic resonance imaging
T3: triiodothyronine
T4: thyroxine
TFTs: thyroid function tests
TSH: thyroid stimulating hormone

## Declarations

### Copyright

© The Author(s) 2024.
